# Generation and Characterization of a DNA-GCN4 Oligonucleotide-Peptide Conjugate: The Impact DNA/Protein Interactions on the Sensitization of DNA

**DOI:** 10.3390/molecules25163630

**Published:** 2020-08-10

**Authors:** Paweł Wityk, Rafał Piątek, Robert Nowak, Dorota Kostrzewa-Nowak

**Affiliations:** 1 Faculty of Chemistry, University of Gdańsk, Wita Stwosza 63, 80-308 Gdańsk, Poland; 2 Faculty of Chemistry, Gdańsk University of Technology, Narutowicza 11/12, 80-233 Gdańsk, Poland; rafal.piatek@pg.edu.pl; 3 Centre for Human Structural and Functional Research, University of Szczecin, 17C Narutowicza St., 70-240 Szczecin, Poland; robert.nowak@usz.edu.pl (R.N.); dorota.kostrzewa-nowak@usz.edu.pl (D.K.-N.)

**Keywords:** radiotherapy, sensitizers, DNA-protein interactions

## Abstract

Radiotherapy, the most common therapy for the treatment of solid tumors, exerts its effects by inducing DNA damage. To fully understand the extent and nature of this damage, DNA models that mimic the in vivo situation should be utilized. In a cellular context, genomic DNA constantly interacts with proteins and these interactions could influence both the primary radical processes (triggered by ionizing radiation) and secondary reactions, ultimately leading to DNA damage. However, this is seldom addressed in the literature. In this work, we propose a general approach to tackle these shortcomings. We synthesized a protein-DNA complex that more closely represents DNA in the physiological environment than oligonucleotides solution itself, while being sufficiently simple to permit further chemical analyses. Using click chemistry, we obtained an oligonucleotide-peptide conjugate, which, if annealed with the complementary oligonucleotide strand, forms a complex that mimics the specific interactions between the GCN4 protein and DNA. The covalent bond connecting the oligonucleotide and peptide constitutes a part of substituted triazole, which forms due to the click reaction between the short peptide corresponding to the specific amino acid sequence of GCN4 protein (yeast transcription factor) and a DNA fragment that is recognized by the protein. DNAse footprinting demonstrated that the part of the DNA fragment that specifically interacts with the peptide in the complex is protected from DNAse activity. Moreover, the thermodynamic characteristics obtained using differential scanning calorimetry (DSC) are consistent with the interaction energies calculated at the level of metadynamics. Thus, we present an efficient approach to generate a well-defined DNA-peptide conjugate that mimics a real DNA-peptide complex. These complexes can be used to investigate DNA damage under conditions very similar to those present in the cell.

## 1. Introduction

The radio- and photodegradation of DNA is typically studied using simple molecular models such as nucleobases, nucleotides or short fragments of single- or double-stranded DNA (ssDNA or dsDNA) [1]. However, in vivo, genomic DNA interacts with a large number of proteins that are responsible for various processes in living organisms [2,3]. Contacts between amino acids and nucleobases represent a minority (in respect to interaction with backbone) of the total number of DNA-protein interactions but are critical factors for DNA sequence recognition. Such specific interactions are mostly mediated by hydrogen bonding between amino acid side chains and nucleobases [4]. The most abundant of these interactions are between guanine bases and arginine/lysine residues, which are present almost in all classes of proteins that interact with DNA [5,6,7].

Specific interactions within DNA-protein complexes are likely to influence DNA damage induced by UV or gamma radiation. Indeed, the presence of proton-donating or proton-accepting groups in the vicinity of native or modified DNA bases changes their electron affinity and spectral properties. As shown by Schaefer et al., nucleobases in the gas phase produce only weak dipole bonds or resonance anions [8]. However, the presence of other molecules, such as amino acids, stabilizes the valence anions of nucleobases. In the case of proton donating groups, for example, the amino acids lysine or arginine, electron attachment to nucleobase could induce barrier-free proton transfer, which produces stable valence anions [9].

Quantum chemical calculations predict that interactions between amino acid side chains and adenine-thymine (AT) Watson-Crick base pairs lower the radical transfer barrier from adenine to the sugar-phosphate backbone, increasing the rate of DNA damage. In addition, specific interactions between lysine or arginine and guanine in guanine-cytosine (GC) base pairs should decrease the rate of electron transfer to the DNA backbone [10]. Solumn et al. showed a statistically significant reduction in the number of breaks protein-bound DNA in comparison with DNA that was not interacting with protein [11]. However, Sanche et al. suggested that interaction with the arginine system leads to the increase of DNA damage [12]. Furthermore, proteins interacting with DNA could shield oligonucleotides from radicals produced during irradiation [13].

Protein-DNA complexes also change the physico-chemical properties of each component. Specifically, the photosensitivity of the oligonucleotide will be changed. Strong interactions will form tight structures, changing the conformation of the nucleic acid. For example, previous reports have indicated that the nucleic acid could bend or wrap around the protein, influencing photoirradiation-triggered electron transfer rates along DNA [14,15].

A well-defined theoretical and experimental model system is necessary to study the influence of protein-DNA interactions on DNA damage. The system should be simple enough to permit both instrumental analysis and degradation mechanism prediction. The basic fragment of the GCN4 protein [16] could fulfill these criteria. The amino acids in GCN4 participate in specific interactions with all four nucleobases, as well as carrying out shielding of DNA from radicals [13]. However, oligonucleotide analysis in fragments longer than 100 bp is challenging [17,18]. Therefore, the DNA fragment should be short and consist of only one binding sequence for GCN4. To satisfy this requirement, the protein GCRE (5′-TGACTC-3′) consensus half-site was selected [19,20]. The GCN4 protein binding mechanism has been reported by many research groups [21]. Nevertheless, the reduction of the length of the protein (to include only the interacting region) requires the covalent bonding of DNA with the peptide to preserve the inherent stability of the secondary structure of the complex system as well as maintaining the binding constant [21]. Finally, a suitable method is required to maintain mild conditions and high reaction efficiency. One such method is the click chemistry reaction of Huisgen cycloaddition, which has been employed by many research groups [22,23].

In the current work, we studied the physicochemical properties of a dsDNA/peptide complex, which mimicked the interaction between the GCN4 protein and the GCRE consensus half-site (5′-GTCATC-3′). The thermodynamic properties such as ΔG and ΔH of peptide binding and the melting temperature of the system were measured. These findings were then juxtaposed with the theoretical estimates of binding free energy obtained by metadynamics. Additionally, enzymatic digestion (DNase I footprinting) of the DNA-peptide conjugate was characterized by liquid chromatography-mass spectrometry (LC-MS). We theorized that the proposed approach would generate a simple but well-defined, oligonucleotide-peptide complex comprising all the structural elements responsible for specific interactions present in a real DNA-protein system. As mentioned before brominated nucleobases are being investigated as potential cancer treatment drugs [1]. Thus, using the proposed methodology and proposed molecular system, we have synthetized the 8-bromo-2′-deoxyguanosine labeled dsDNA/peptide complex which can be used for further sensitizing radio- or photo- DNA damage studies.

## 2. Results and Discussion

### 2.1. Molecular Properties of the GCN4-DNA Molecular System

In nature, three major protein domains interact with DNA: α-helices, β-sheets or a mixture of both structures (α/β). Most of the specific interactions are mediated by α-helix domains [24]. One of the well-known proteins belonging to this family is GCN4, a eukaryotic transcriptional activator that binds to its target DNA as a dimer (consisting of a leucine zipper and basic fragment motif responsible for binding the major groove of DNA). Thus, this protein can be employed in the construction of a model DNA-protein interaction system. Ideally, such a molecular system should be simple. Therefore, for our model, we selected only a basic fragment of the GCN4 protein. This fragment tightly interacts with DNA. Indeed, as shown previously by Stanojevic & Verdin [21], decreasing the GCN4 protein to its basic fragment should be sufficient to mediate the specific interaction with DNA as in the native protein. However, removing the part of the protein responsible for maintaining the stable α-helix complex with DNA (the leucine zipper domain) will decrease the DNA binding constant of the peptide and could disrupt the secondary or tertiary structure [20]. To remedy this, we mimicked the missing part of the protein by covalently linking the basic fragment of the GCN4 protein with one of the DNA strands, ensuring we obtained 1:1 peptide/DNA conjugates.

**Figure 1:**
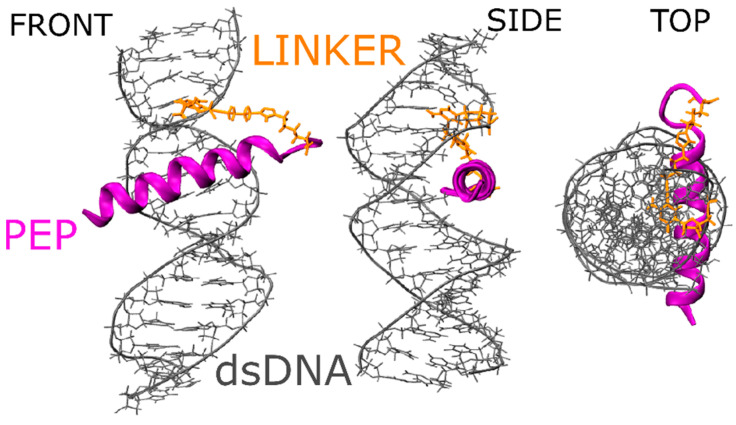
Front, side and top views of the DNA-peptide conjugate in double-stranded DNA form after click chemistry reaction; purple—peptide in α-helix conformation, gray—dsDNA in B-DNA form and orange—azide-alkyne Huisgen cycloaddition linker.

We required a gentle but efficient mechanism of covalently linking DNA with peptide molecules. After a comprehensive literature search, we selected and employed the click chemistry reaction protocol of azide-alkyne cycloaddition to obtain conjugates. Many research groups have demonstrated that this method is efficient at joining biomolecules present at low concentrations. More importantly, selecting an appropriate merging method allows us to design the molecular system with the use of computational methods before carrying out the expensive and time-consuming synthesis of conjugates [23]. Accordingly, we used Molecular Dynamics to determine that we should replace the leucine-zipper part of the protein by attaching the basic fragment of GCN4 protein (PEP: H-ASP-PRO-ALA-ALA-LEU-LYS-ARG-ALA-ARG-ASN-THR-GLU-ALA-ALA-ARG-ARG-SER-ARG-ALA-ARG-LYS-GLY-GLY-LYS(N3)-NH2) to one of the nucleobases of the target DNA molecule—cytosine (C8-Alkyne dC)—for detailed structure of linker please see Figure S1 in the Supplementary Materials. In addition, to allow full flexibility of peptide (PEP), we added the glycine linker (GLY-GLY) prior to the connection of the peptide with cytosine (loop fragment in the “top view,” Figure 1).

Finally, during the molecular dynamics, we were able to analyze the hydrogen bonding pattern (Figure S2 in Supplementary Materials) between amino acids and the DNA interacting fragment G-ARG, C-ASN, as well as hydrophobic interactions like T-ALA. These findings are in agreement with native protein PDB structures (see Section 3).

### 2.2. Efficient Synthesis of a ssDNA/Protein Complex

In order to synthetize the DNA/peptide system and perform structure and stability studies of proposed system, we have prepared 4 different double stranded oligonucleotides mixtures: dsDNA, dsDNA*, dsDNA*-PEP and dsDNA**-PEP, native (dsDNA, dsDNA-PEP) and labeled (dsDNA*, dsDNA**-PEP) by 8-bromo-2′-deoxyguanosine (Y). All of the sequences are shown in Table 1. Reducing the GCN4 protein to short peptide (PEP) and maintaining the structure (e.g., hydrogen bonding pattern between amino acids and nucleobases) of this fragment requires use of the linker between oligonucleotide and peptide. To accomplish this we have used the CLICK chemistry reaction protocol and were able to synthetize the ssDNA*-PEP and ssDNA**-PEP molecules. Finally, after annealing of ssDNA*-PEP and ssDNA**-PEP with ssDNA B we received dsDNA*, dsDNA**-PEP systems which are mimicking the native GCN4/DNA interactions.

**Figure 2:**
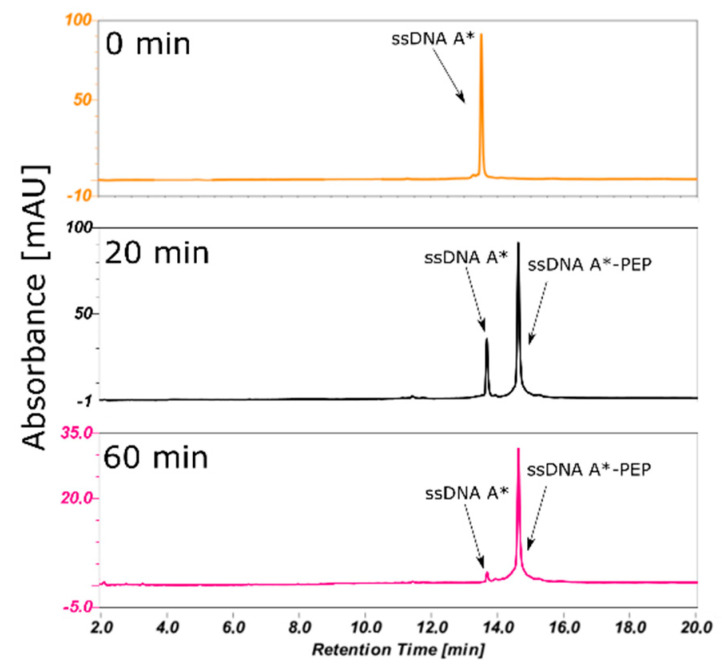
Progression if the click chemistry reaction monitored by high-performance liquid chromatography (HPLC), after 20 (black) and 60 (pink) mins. ssDNA (A*) standard before the reaction is shown in orange. After 60 min all of the substrate (ssDNA A*) was transformed into product (ssDNA A*-PEP). Injection volumes was as follows: 10 µL, 10 µL and 5 µL respectively for 0, 20 and 60 min. HPLC conditions: XBridge OST reverse-phase C18 column; mobile phases A: 50 mM triethylamine acetate (TEAA) and 1% acetonitrile in water, B: 80% ACN in water; a gradient of 0–20%/20 min B was used, maintaining 1 mL/min flow rate; recorded at 260 nm.

The Huisgen click chemistry reaction involves Cu^+^ ions as reaction catalyst (maintained by CuSO_4_ and ascorbic acid) as well as DNA by modified a triple bond and N_3_ labeled peptide. The conditions of such a redox reaction—Cu^2+^/Cu^+^—could damage the biomolecules. In order to increase the efficiency, we have used Tris(3-hydroxypropyltriazolylmethyl)amine (THPTA), a stabilizing ligand which complexes with Cu^+^ cations, reducing the likelihood of DNA damage and the time of the reaction [25]. Additionally, hydroxyl radicals are also formed during the reaction. To scavenge these radicals, we used t-butanol was used [26]. In addition, to decrease components reacting with oxygen, the mixture was purged with argon by 5 min (min) [27]. These precautions allowed us to obtain a protein/DNA complex at a much faster rate than reported by others; 1h at room temperature (300 K) with minimal loss of biomolecules [23]. Monitoring the progress of the reaction (Figure 2) allowed us to select the best time to stop the reaction by adding Ethylenediaminetetraacetic acid (EDTA) into the mixture, preventing unnecessary loss of the material. After high-performance liquid chromatography (HPLC) purification followed by lyophilization, the efficiency of the reaction and following purification was estimated as 90% by weighting the powder of the substrate/product (in terms of moles of ssDNA (A*) or ssDNA(A**) substrate to moles of product).

### 2.3. Annealing of ssDNA into dsDNA

**Figure 3:**
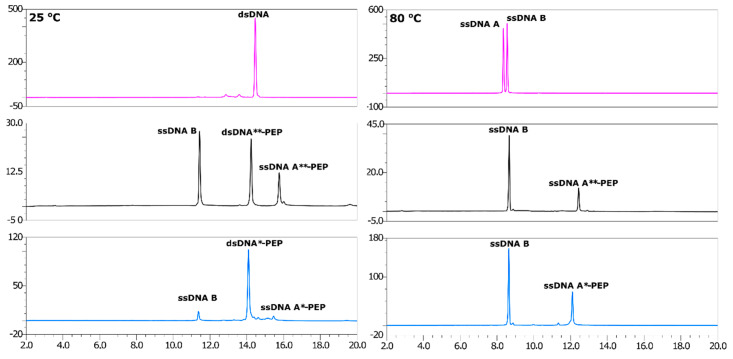
HPLC (**left**) and denaturative HPLC (dHPLC)—(**right**) analysis of the dsDNA product obtained after annealing ssDNA peptide conjugates. Purple—non-labeled dsDNA, black—dsDNA**-PEP, blue—dsDNA*-PEP. HPLC conditions: XBridge OST reverse-phase C18 column; mobile phases A: 50 mM triethylamine acetate (TEAA) and 1% acetonitrile in water, B: 80% ACN in water; a gradient of 0–20%/20 min B was used, maintaining 1 mL/min flow rate; recorded at 260 nm. The picture clearly presents the high purity of obtained double stranded DNA systems—almost no other peaks visible.

**Figure 4:**
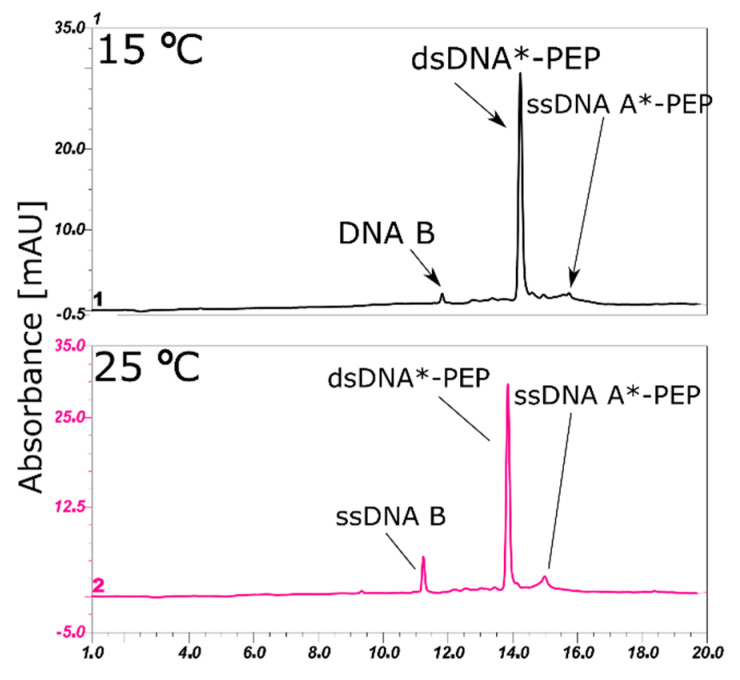
HPLC analysis of the dsDNA*-PEP product obtained after annealing ssDNA A*-PEP peptide conjugates, showing a decrease in the ssDNA form at a lower temperature suggesting the interaction between ssDNA B complementary strand do ssDNA A*-PEP. HPLC conditions: XBridge OST reverse-phase C18 column; mobile phases A: 50 mM triethylamine acetate (TEAA) and 1% acetonitrile in water, B: 80% ACN in water; a gradient of 0–20%/20 min B was used, maintaining 1 mL/min flow rate; recorded at 260 nm.

The annealing reaction of double-stranded DNA is relatively easy to predict. However, choosing the best temperature to obtain B-DNA, as well as the α-helix conformation of the peptide, is challenging. A lower temperature (80 °C) of strand dissociation was used and the annealing temperature was set to 55 °C (see DSC results), which is the temperature of peptide melting. The association of ssDNA A, ssDNA A*-PEP, ssDNA A**-PEP and ssDNA B were confirmed by HPLC analysis (Figure 3 and Figure 4). The lower HPLC signals of ssDNA A**-PEP and ssDNA A*-PEP are due to different extinction coefficients for native and labeled DNA strands. The difference in extinction coefficients is due to the fact that the: (i) conformation of ssDNA A is different than ssDNA A*-PEP and ssDNA A**-PEP, (ii) labeled oligonucleotides consist of additional 1,2,3-triazole moiety, bromine and amino acids which shifts the maximum of absorbance from around 260 nm towards higher wavelengths.

**Figure 5:**
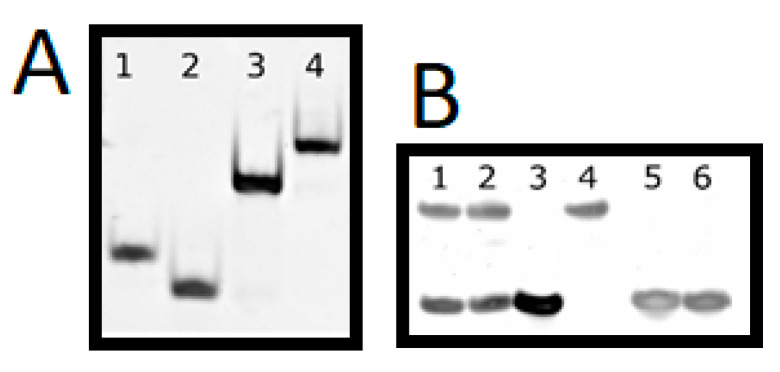
(**A**)—Native polyacrylamide gel electrophoresis (PAGE) of biomolecules. Lane: (1)—ssDNA* A, (2)—ssDNA B, (3)—dsDNA, (4)—dsDNA*-PEP. (**B**)—Denaturing PAGE of biomolecules. Lane: (1)—dsDNA**-PEP, (2)—dsDNA*-PEP, (3)—dsDNA, (4)—ssDNA*-PEP, (5)—ssDNA B, (6)—ssDNA* A. PAGE conditions: 15% PAGE with or without 7M urea; 150 V for 1 or 1.5 h for native and denaturating PAGE, respectively, stained with SYBR.

In addition to HPLC analysis PAGE (Figure 5) was performed to analyze strand hybridization and check of the purity of dsDNA, dsDNA*-PEP and dsDNA**-PEP conjugates. It is important to note that ssDNA A*-PEP and ssDNA**-PEP possess a different charge than ssDNA A. This is due to the covalently attached peptide which is positively charged (ARG and LYS, owing to a total charge of +5) and thus migrates slowly.

### 2.4. LC-MS Analysis of dsDNA-Peptide Conjugate

**Figure 6:**
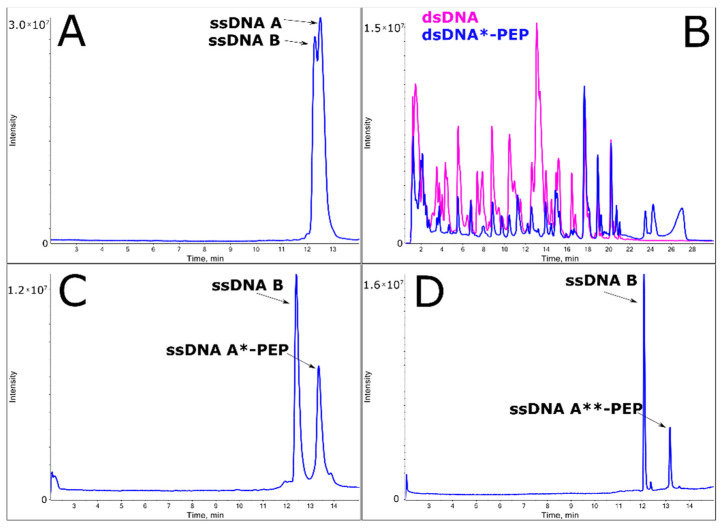
Liquid chromatography-mass spectrometry (LC-MS) analysis of the following biomolecules represented by Total Ion chromatogram: (**A**) dsDNA; (**B**) DNase I digestion patterns of dsDNA (pink) and dsDNA*-PEP (blue); (**C**) dsDNA*-PEP; (**D**) dsDNA**-PEP. The mobile phase A consisted of 400 mM HFIP and 15mM TEA in deionized ultrapure water and mobile phase B consisted of 200 mM HFIP, 7.5 mM TEA 50% (*v*) MeOH and 50% (*v*) H_2_O. The flow gradient condition was as follows: for purity check, 0–40% B at 15 min at 85 °C, 0–1 min 0% B; for footprinting analysis, 1–30 min 60% B at 50 °C.

Additionally, purity and receiving the desired product was confirmed by LC-MS analysis. One of the most demanding techniques for investigating DNA molecules is high-resolution LC-MS (see Table 1 for the calculated and experimental masses of all oligonucleotides). Here, we present a method for qualitative and quantitative analysis of oligonucleotides and peptide-DNA conjugates. It is known that without special additives to the mobile phase, oligonucleotides are poorly ionized by electrospray ionization (ESI). Therefore, to increase the efficiency of this process—which leads to an increase in the total ion current of the oligonucleotide—HFIP (hexafluoroisopropanol) and TEA (triethylamine) were used to obtain the high selectivity of the LC separation technique, even for the DNase “sequencing/footprinting” reaction. Notably, oligonucleotides ionize in negative ionization mode; however, peptides tend to ionize in positive ionization mode. Thus, obtaining good spectra for oligonucleotide-peptide conjugates (Figure 6, Figure S3) challenging. Nevertheless, using specially designed LC-MS conditions (see Materials and Methods), we were able to analyze dsDNA-peptide conjugates robustly. The reliability of such an analysis is crucial when analyzing DNA or peptide damage. LC-MS analysis confirmed the high purity of dsDNA*-PEP and dsDNA**-PEP solutions, the later one is crucial for the photo- and radio- damage studies. The bromine in guanosine moiety is responsible for the sensitivity of oligonucleotide on high energy photons or solvated electrons in DNA damage studies upon sensitization. Indeed, during the CLICK chemistry reaction the bromine could lead to DNA damage and lower yields of labeled product, thus mass spectra results confirmed that we were able to synthetize “fragile” ssDNA**-PEP molecule. In addition, as in case of HPLC signals and different extinction coefficients, observing the mass spectra, we can see that the lower signal for ssDNA*-PEP and ssDNA**-PEP than ssDNA B. We can observe this phenomenon because the ssDNA*-PEP and ssDNA**-PEP are attached to positively charged peptide, when oligonucleotide is negatively charged and we are recording the data in negative ionization mode (Figure 6, pane: A, C, D).

### 2.5. DNase I Footprinting Reveals the Binding Pattern of DNA and Peptide

**Figure 7:**
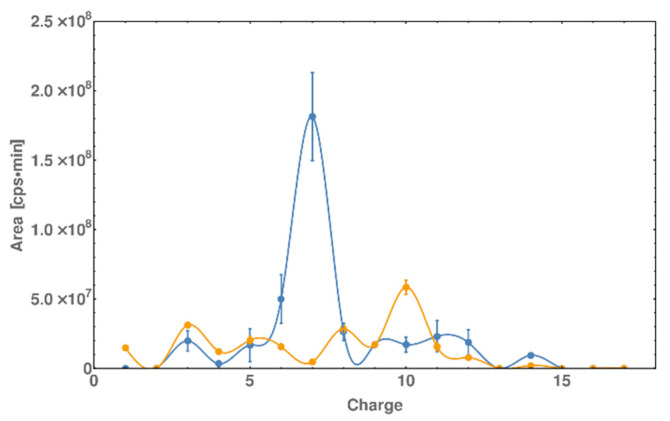
Digestion pattern of dsDNA (blue) and dsDNA*-PEP (orange) by DNase I [Charge axis correspond to the following sequence starting from 5′ end—ssDNA* A (5′-GCA CGT CAT CCG TATAG-3′)]. The resulting chromatogram (based on data similar to the PAGE method) is taken from LC-MS analysis. Each peak on TIC (total ion current) was identified and their areas were plotted against the charge of the recognized fragment. The AP-1 sequence corresponds to the 5 to 8 charge fragment. 200 pmol of dsDNA*-PEP or dsDNA was incubated with 0.04 U of DNase I in 10 mM CaCl_2_, 100 mM MgCl_2_, 100 mM Tris-HCl (pH 8.9) buffer solution. The digestion was performed at 4 °C for 10 min.

One of the crucial features of protein/DNA interactions is the presence of a specific protein binding site present in DNA. It has been previously shown that the GCN4 protein interacts with DNA through the AP-1 binding site [28]. To confirm that our complex behaves in the same way, we performed a DNase I footprinting assay. However, to streamline the procedure of footprinting, we avoided the use of phosphorous labeling or other sensitive and time-consuming methods. We propose footprinting with use of HPLC and high-resolution mass spectrometry (Figure 6B) to perform qualitative and quantitative analysis of all fragments. High-resolution spectrometry allowed us to carefully determine all possible fragments obtained by digestion and generated a simulation of the gel electrophoresis patterns (Figure 7). The dsDNA and dsDNA*-PEP were digested by DNase at 4 °C. The resulting identified oligonucleotides fragments by means of LC-MS were listed in Supplementary Table S1. The identified fragments were reported as parts of whole ssDNA A and ssDNA B strands. After the identification, the quantification of all fragments were conducted as the area of individual peaks (reported in Supplementary Table S1). After identification the area of peaks were juxtaposed with the charge of the fragment and drawn as a graph (see Figure 7). As indicated we can see the maximum around charge 7 (blue line, Figure 7) which represent the most abundant cleavage of oligonucleotide by DNase. This part of the oligonucleotide represent the recognition place of GCN4 protein or our peptide fragment (AP-1). In case of dsDNA we can see the maximum which cannot be seen for the dsDNA*-PEP complex, thus we can conclude that the peptide is blocking the access for DNase.

### 2.6. Circular Dichroism (CD) Spectra Measurement

**Figure 8:**
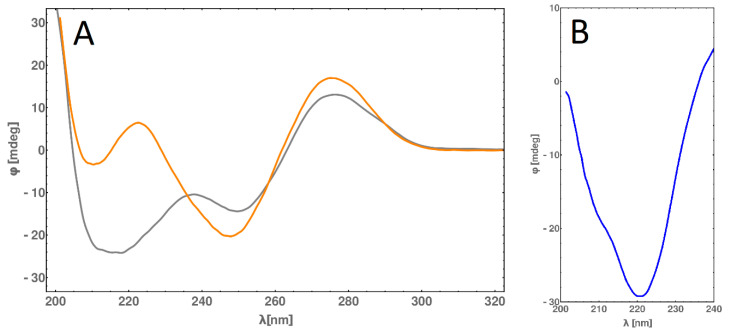
(**A**)—Circular dichroism spectra obtained for dsDNA (in orange) and dsDNA*-PEP (gray) at 25 °C. (**B**)—The “difference” circular dichroism spectrum obtained for dsDNA*-PEP at 25 °C. The resulting spectrum represents the difference between dsDNA*-PEP and dsDNA CD spectra, rescaled by the concentration of the sample. Far-UV CD was measured on a Jasco J-810 spectropolarimeter (Jasco, Tokyo, Japan). Experiments were performed in 100 mM TBA buffer, pH 8 in 1 mm pathlength cuvette. The sample concentrations were 0.25 and 0.4 mg/mL for dsDNA-PEP and dsDNA, respectively. Spectra were recorded from 190 to 350 nm with a 1 nm step size.

The selected linking and annealing conditions allowed us to obtain a B-DNA complex linked with a-helix peptide, confirmed by circular dichroism (CD) measurements. The curvature (shape) of B for dsDNA is well known, so it is easy to compare our results to the results for native DNA. However, the interpretation of CD spectra of the dsDNA-peptide conjugate is disrupted by the interaction of DNA with the peptide; the simple addition of DNA and peptide contribution could not generate the proper CD spectra of the dsDNA-peptide complex. Nevertheless, subtracting the CD spectra values of DNA from DNA-PEP allowed us to obtain a “difference” CD spectra (Figure 8, pane A) and observe the peptide structure. In Figure 8, pane B, the characteristic pattern of α-helix conformation, the “v” shape with two characteristic minima of n-π* and π-π* transition, can be observed. We conclude that the peptide forms an α-helix conformation as its secondary structure. Furthermore, CD spectroscopy allowed us to obtain the melting temperature of the α-helix peptide (64 °C) interacting with DNA by extracting the changes at 220 nm wavelength, this changes correspond to changes in peptide conformation (the peptide does not have any aromatic amino acids).

### 2.7. DSC Melting Profiles

**Figure 9:**
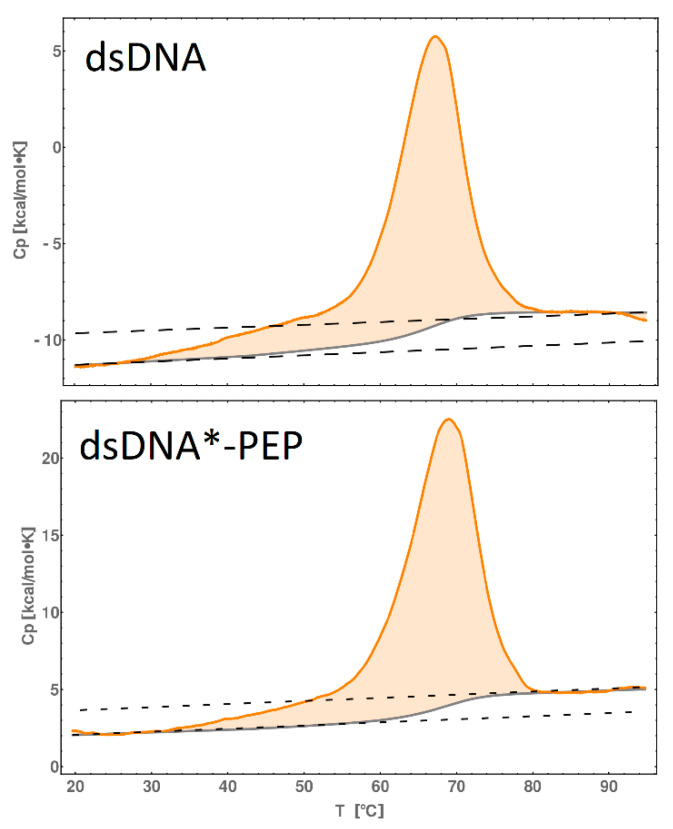
Thermograms for dsDNA and dsDNA*-PEP (orange line) and baseline (gray line) of dsDNA and dsDNA-PEP in 100 mM PBS solution. Dashed lines indicate the heat capacity difference between folded and unfolded states. The DSC spectra after the sigmoidal baseline subtraction are shown in supplementary material (Figures S3 and S4). DSC experiments were performed on the CSC 6300 Nano-DSC III microcalorimeter (Calorimetry Sciences Corporation, Lindon, UT, USA) with the capillary cell volume of 0.299 mL at a temperature range of 0 to 90 °C. DSCRun software (Calorimetry Sciences Corporation, Lindon, UT, USA) was used to record the data. The concentration of dsDNA (molecular mass 10.38 kDa) and dsDNA*-PEP (molecular mass 13.15 kDa) were 0.466 and 0.326 mg/mL, respectively, in each experiment. The analysis was performed with a scanning rate of 1 °C·min^−1^.

**Figure 10:**
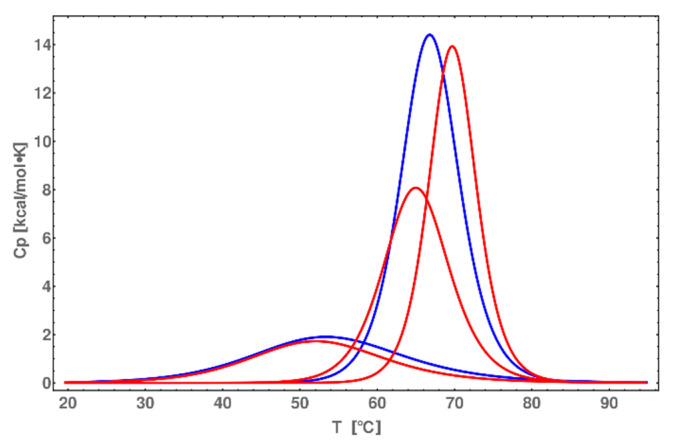
Comparison of deconvoluted thermograms of dsDNA (blue) and dsDNA*-PEP (red).

Investigating the thermodynamic properties of a DNA-peptide complex can be challenging. In order to perform reliable measurements of the ΔG, ΔH or ΔS of peptide binding to DNA, a large quantity of the complex is needed. Using a high concentration of dsDNA*-PEP complex as well dsDNA, we were able to perform nano-DSC measurements and calculate the thermodynamic parameters of “dissociation/melting” processes. Figure 9 shows the thermogram of dsDNA, where the dashed lines represent the extrapolated C_p_ values for the double-stranded state and single-stranded state (higher temperatures). This extrapolation comes from the fact that the dsDNA ends are relaxing/bending during heating. Thus, the melting temperature of dsDNA is T_m,DNA_ = 66.8 °C, ΔC_p_ ~ 2 kcal/mol, the orange space represents the free energy, ΔH ~ 183 kcal/mol and the solid gray line represents the sigmoidal shaped baseline. Deconvolution of the heat capacity profile (Figure 10) allowed us to distinguish between two separate contributions: the DNA ends relaxing and melting of DNA (disruptors of the Watson-Crick pattern), which are consistent with previous findings [29]. For the dsDNA*-PEP complex (Figure 9), the melting temperature increased to T_m,PEP_ = 68.8 °C, ΔC_p_ ~ 1.4 kcal/mol, ΔH ~ 246 kcal/mol. Thus, using the temperature-dependent equations of thermodynamic functions $\Delta H\left(T\right)=\Delta H_{M}+\Delta \mathrm{Cp}\left(T-T_{M}\right)$, $\Delta S\left(T\right)=\Delta S_{M}+\Delta \mathrm{Cp}\Delta \mathrm{Ln}\left(T/T_{M}\right)$, where T_M_, ΔH_M_ and ΔS_M_ are the thermodynamic function in melting temperature point, we can obtain temperature-dependent functions (Table 2) for 298 K.

It is worth noting that the values for DNA melting are consistent with different DNA melting energies. A simple subtraction of ΔG_DNA_ from ΔG_DNA-PEP_ produces the estimated 10 kcal/mol sum of interaction and melting energy of the peptide. It is only impossible to strictly obtain interaction or melting energy because the ssDNA is covalently linked with peptide. A comparison of deconvoluted heat capacity function (Figure 10) allows us to propose that the peptide is melting cooperatively with DNA, changing the melting temperature of DNA by 2 °C (Gaussian with the highest center of temperature). The deconvolution of heat capacity function was performed to distinguish between contributions: (i) dsDNA ends melting (the lowest melting temperature), (ii) peptide melting (middle around 64 °C), (iii) core dsDNA melting (the highest melting temperature). Taking into account the fact that the peptide (PEP) which we are using is only a part of the protein GCN4 and the peptide itself exist in solution as random coil, we should observe the cooperative transition, because peptide in α-helix form cannot exist without dsDNA. The deconvolution of heat capacity function after subtraction of the sigmoidal baseline was performed for 2 or 3 Gaussian functions for dsDNA and dsDNA*-PEP, respectively (Figure 10). These should correspond to the melting of the peptide; the heat (the area of Gaussian) is ~8 kcal/mol. There is a strong similarity in one Gaussian function between the two systems, shifted in melting temperature (maximum of the Gaussian function). However, we can extract the data of peptide melting with the help of circular dichroism spectroscopy, which gives us insights into the physical properties such as peptide melting temperature.

### 2.8. CD Melting Temperature Comparative Analysis of dsDNA and dsDNA-PEP

**Figure 11:**
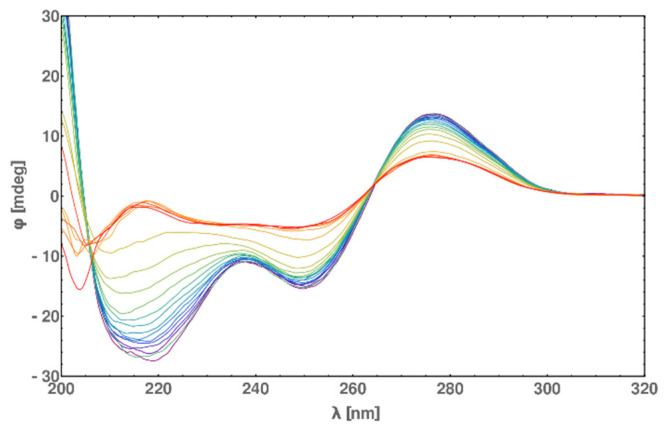
Circular dichroism spectra of dsDNA*-PEP over 0–90 °C temperature range in 5 °C increments from 0 °C (blue) to 90 °C (red). As we can see the melting of the peptide and dsDNA can be seen by the 220 nm ellipticity increasing with the increasing temperature allowing for the determination of melting temperature ~64 °C of the α-helix. Studies of the melting of dsDNA alone can be predicted by monitoring the 280 nm ellipticity—thus we can see the cooperativity of the DNA and peptide melting process.

**Figure 12:**
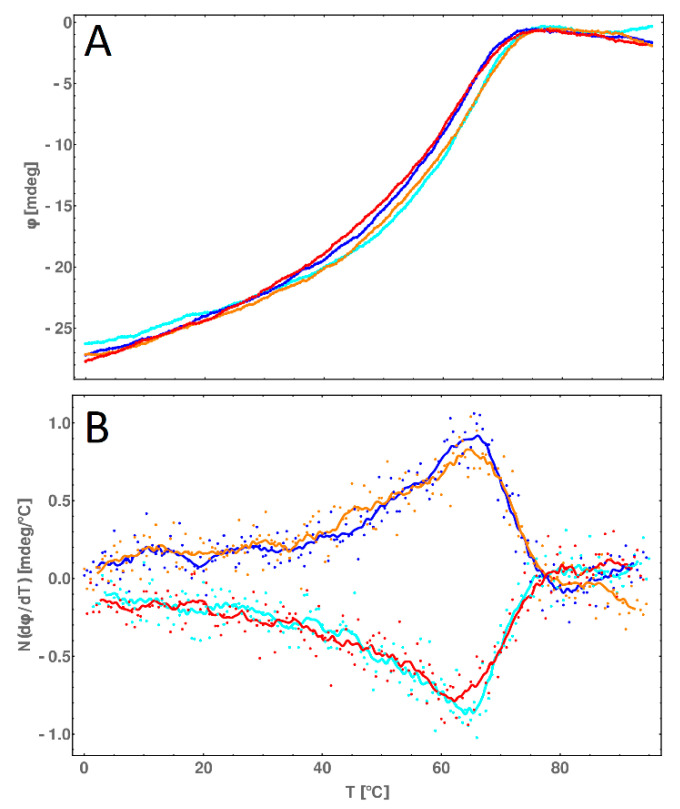
(**A**) Circular dichroism melting profiles (recorded at 220 nm) of dsDNA*-PEP over a 0–90 °C temperature range. (**B**) Circular dichroism melting profiles (recorded at 220 nm) of dsDNA*-PEP over a 0–90 °C temperature range. Heating (orange and blue curves) and cooling (red and cyan curves) experiments are shown. Curves represent the moving average of respective color dots (real measurement). Far-UV CD was measured on a Jasco J-810 spectropolarimeter (Jasco, Tokyo, Japan). Different measurements (for both heating and cooling) were conducted at a 1 °C/min heating/cooling rate from 0 to 90 °C or 90 to 0 °C and normalized against TBA buffer.

The circular dichroism measurements of dsDNA-PEP in different temperatures ranging from 0 to 90 °C allows us to verify our assumption from DSC measurement that the peptide is melting cooperatively with the DNA. In Figure 11, the shifts of circular dichroism values are decreasing in both regions of 200–220 nm and 240–260 nm (indicating α-helix and B-DNA, respectively) in the same manner. Additionally, we were able to find the melting temperature of the α-helix using the 220 nm wavelength (Figure 12, pane A) and then differentiate (Figure 12, pane B) the circular dichroism value. In this way, we obtained a melting temperature of 64 °C, which is in excellent agreement with the values obtained by DSC measurements. The melting experiments clearly presents that the process of melting of dsDNA*-PEP complex is reversible because the heating and cooling curves are almost identical (Figure 12, pane A).

### 2.9. Metadynamics of dsDNA-PEP Complex

**Figure 13:**
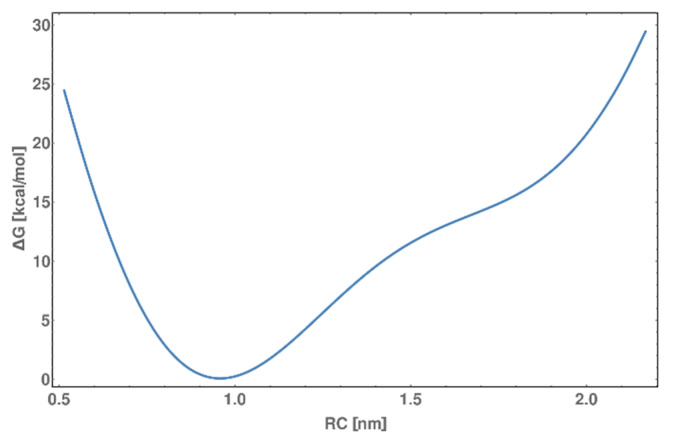
Free energy profile of peptide binding to DNA obtained by a well-tempered metadynamics scheme over 5 µs simulation. The reaction coordinate (RC) represents the distance between the centers of masses of the peptide and the AP-1 interacting site. The bound state is located around 0.9 nm. The calculated binding energy of α-helix to DNA is calculated to be ~10 kcal/mol.

Finally, to better understand the behavior of the model system, well-tempered metadynamics were performed. This analysis allowed us to obtain the binding energy of the α-helix to DNA. The calculated energy is ~10 kcal/mol (see Figure 13) according to density integration (assuming 0.9 nm the as bound state and 1.5 nm as the dissociated state). The DSC measurement revealed the ~10 kcal/mol melting and interaction energy. However, this experimental energy is not the direct sum of interaction energy and melting energies due to covalently linked biomolecules. Thus, the true energy of the α-helix interaction with DNA will be higher but is challenging to obtain. The results clearly indicate that the predicted theoretical structures corresponds to experimentally obtained, allowing for more theoretical investigations.

## 3. Materials and Methods

### 3.1. Materials

Native and labeled (8-Bromo-2′-deoxyguanosine and C8-Alkyne-dC) oligonucleotides were purchased from Metabion (Planegg, Germany). ssDNA A: 5′-GCA CGT CAT CCG TATAG-3′, ssDNA A*: 5′-GCA XGT CAT CCG TATAG-3′ (X = C8-Alkyne dC), ssDNA A**: 5′-GCA XYT CAT CCG TATAG-3′ (X = C8-Alkyne dC Y = 8-bromo-2′-deoxyguanosine) ssDNA B: 5′-CTA TAC GGA TGA CGT GC-3′. Azine-modified azide moiety peptide was purchased from GeneCust (Boynes, France)—PEP: H-ASP-PRO-ALA-ALA-LEU-LYS-ARG-ALA-ARG-ASN-THR-GLU-ALA-ALA-ARG-ARG-SER-ARG-ALA-ARG-LYS-GLY-GLY-LYS(N3)-NH_2_; All oligonucleotides and peptides were HPLC purified. Ascorbic acid (anhydrous, ≥99.99%), CuSO_4_ (anhydrous, powder, ≥99.99%), Aminoguanidine hydrochloride (≥98%), THPTA: Tris(3-hydroxypropyltriazolylmethyl)amine (≥95%), Tris Buffered Saline (pH 8, powder), DNase I (Type II, from Bovine), Acetonitrile (LC-MS grade), Methanol (LC-MS grade), 1,1,1,3,3,3-Hexafluoro-2-propanol (HFIP, LC-MS grade), CaCl_2_ (1M stock solution) and MgCl_2_ (1M stock solution) were purchased from Sigma Aldrich (Warsaw, Poland). In all experiments, ultrapure water was used (Merck Millipore system, Darmstadt, Germany).

### 3.2. HPLC

HPLC and DHPLC were carried out using XBridge OST, reverse-phase C18 column (2.5 µm in particle size, 4.6 × 50 mm); mobile phases A: 50 mM triethylamine acetate (TEAA) and 1% acetonitrile (can) in water, B: 80% ACN in water. For purification and material verification, a gradient of 0–20%B at 25 °C or (80 °C for DHPLC) was used, maintaining 1 mL/min flow rate. Separation was performed on a DionexUltiMate 3000 System with Diode Array Detector monitoring at 260 nm wavelength.

### 3.3. LC-MS

LC-MS analysis of DNA fragments was carried out using the Ultra High Performance Liquid Chromatography (UHPLC, Nexera X2, Kioto, Japan) coupled to a mass spectrometer TripleTOF 5600+ (AB SCIEX, Concord, Canada) equipped with a duo-electrospray interface operated in negative ionization mode. Acquity UPLC BEH C18 1.0 × 50 mm column (Waters, Milford, CT, USA) with flow rate maintained at 0.1 mL/min was used for chromatographic separation of oligonucleotides or conjugates. The samples were in situ desalted on column by effluent diverting to waste for 1 min after injection during each analysis. The mobile phase A consisted of 400 mM HFIP and 15 mM TEA in deionized ultrapure water and mobile phase B consisted of 200 mM HFIP, 7.5 mM TEA 50% (*v*) MeOH and 50% (*v*) H_2_O. The flow gradient condition was as follows: for purity check, 0 to 40% B at 15 min at 85 °C, 0–1 min 0%B; for footprinting analysis, 1 to 30 min 60% B at 50 °C. MS operation parameters for negative ionization mode (in all experiments) were as follows: spray voltage −4.5 kV, nebulizer gas (N_2_) pressure 30 psi, collision energy at −10 V, declustering potential −150V and the source temperature was maintained at 300 °C

### 3.4. Native and Denaturing PAGE Electrophoresis

Denaturative PAGE—15% denaturing polyacrylamide gels were prepared in 1 × TBE buffer and 7M urea. Native PAGE—15% polyacrylamide gels were prepared in 1 × TBE buffer. The gel was electrophoresed at 150 V for 1 or 1.5 h for native and denaturating PAGE, respectively. The gel was visualized after staining with Sybr Gold (5 uL stock solution (10,000× concentrated) into 50 mL of ultrapure water) using a Fusion FX imaging system (VilberLourmat, Munich, Germany).

### 3.5. DNase Footprinting

Two hundred pmol of dsDNA*-PEP or dsDNA was incubated with 0.04 U of DNase I in 10 mM CaCl_2_, 100 mM MgCl_2_, 100 mM Tris-HCl (pH 8.9) buffer solution. The digestion was performed at 4 °C for 10 min and then the solution was directly injected into the LC/MS system for further analysis. Six independent digestion experiments were performed.

### 3.6. Annealing and DNA-Peptide Ligation

To ligate ssDNA A* or ssDNA** with PEP, a click chemistry reaction was performed. After 1 h incubation of the mixed solution of CuSO_4_ (20 mM, 1.25 µL) with THPTA (50 mM, 2.5 µL), oligonucleotide (ssDNA A* or ssDNA**, 12.5 nmol) as well as tert-butanol (2.5 µL), TBS (1 M, 2.5 µL), aminoguanidine hydrochloride (100 mM, 12.5 µL) and peptide (PEP, 25 nmol) were added to the mixture and incubated at room temperature for 10 min. To start the ligation reaction, 12.5 µL of freshly prepared (100 mM stock solution) ascorbic acid was added. The whole mixture was gently stirred, centrifuged, purged with argon (3 min) and incubated at 25 °C for 1 h. Conjugates were purified using HPLC method (Figure 3) and then lyophilized to white powder. Annealing was performed using an Eppendorf thermocycler; equal amounts of lyophilized ssDNA B and ssDNA A* or ssDNA A** were resuspended in 100 mM TBS, mixed in 0.5 mL Eppendorf tubes to a final concentration of 40 uM. The temperature program was as follows: 5 min at 80 °C, 5 min at 55 °C, 30 min at 4 °C. The annealed product was observed by HPLC (Figure 3).

### 3.7. Circular Dichroism (CD) Measurements

Far-UV CD was measured on a Jasco J-810 spectropolarimeter (Jasco, Tokyo, Japan). Experiments were performed in 100 mM TBA buffer, pH 8 in 1 mm pathlength cuvette. The sample concentrations were 0.25 and 0.4 mg/mL for dsDNA-PEP and dsDNA, respectively. Spectra were recorded from 190 to 350 nm with a 1 nm step size. Spectra were averaged from three measurements and normalized against the TBA buffer at different temperatures (0 °C to 70 °C in 10 °C increments). For melting experiments, three different measurements (for both heating and cooling) were conducted at a 1 °C/min heating/cooling rate from 0 to 90 °C or 90 to 0 °C and normalized against TBA buffer. The same samples were used in DSC measurements.

### 3.8. Differential Scanning Calorimetry (DSC) Measurements

DSC experiments were performed on the CSC 6300 Nano-DSC III microcalorimeter (Calorimetry Sciences Corporation, Lindon, UT, USA) with the capillary cell volume of 0.299 mL at a temperature range of 0 to 90 °C. DSCRun software (Calorimetry Sciences Corporation) was used to record the data. The concentration of dsDNA (molecular mass 10.38 kDa) and dsDNA*-PEP (molecular mass 13.15 kDa) were 0.466 and 0.326 mg/mL, respectively, in each experiment. The analysis was performed with a scanning rate of 1 °C·min^−1^. Before the experiment, the cell volume was flushed with 0.1% formic acid and extensively purged with water and 100 mM TBS solution. After several conditioning cycles, during heating, the dsDNA or dsDNA*-PEP degassed solution was injected into a platinum cell. Each heating and cooling experiment was repeated three times and normalized against the buffer.

### 3.9. Molecular Dynamics Structure Preparation

The molecular system consisted of a dsDNA-PEP structure constructed from the basic region (bZIP) of the GCN4 protein and part of the consensus half-site sequence (GCRE) 5′-GTCATC-3.′ Geometries were taken using data from 1YSA (pdb.org). Additional DNA fragments were added with the use of X3DNA software. New residues (linker residues) were parameterized using Gaussian package g09-D.01 at HF/6-31G* level of theory. Protonation states were assigned using the Propka software. The systems were solvated with TIP3P water and neutralized by adding Na^+^ and Cl^−^ to obtain the physiological ionic strength of 0.154 M. The equilibrium MD simulations of the dsDNA-PEP system were performed in a cuboid box with box vectors of around 90 Å in each direction.

### 3.10. Simulation Procedure

All molecular dynamics (MD) simulations were performed using Gromacs 5.01 coupled with PLUMED 2.1 package and AMBER99SB force field with the PARMBSC0 refinement. Periodic boundary condition and Nose-Hover and Berendsen barostat were used for temperature and pressure control, with reference values of 298 K and 1 bar and time constants of 0.1 ps and 2 ps, respectively. The equations of motion were integrated using the leap-frog integration algorithm with a timestep of 2 fs. Equilibration was carried out for 100 ns. This equilibrated structure was used to study the binding properties of the peptide to DNA, with the use of a well-tempered metadynamics scheme. As a reaction coordinate (RC), the distance between centers of masses of peptide and AP-1 sequence was chosen. During the simulation procedure, Gaussians (height 1 kJ/mol and width 0.5 nm, biasfactor—20) were added every 3000 steps over 5 µs of metadynamics. The peptide was maintained with α-helical conformation during sampling by harmonic potential (2 kcal/mol) on carbon atoms.

## 4. Conclusions

We have successfully synthetized and characterized the molecular system consisting of dsDNA and peptide mimicking the native GCN4/DNA interaction. The model system have the same bonding pattern between nucleobases and amino acids, very similar physical and chemical properties to the native GCN4/DNA system. In order to synthesize this remarkable complex, we have decreased the GCN4 transcriptional factor protein to the peptide part responsible only for interacting with DNA. Then, we have chemically synthetized the peptide and oligonucleotide, merge them together by covalent linker and by means of CLICK chemistry to maintain the stability of α-helix of the peptide. This novel approach allows us to finally synthetize the small dsDNA/protein system labeled by 8-bromo-2′-deoxyguanosine, one of the proposed radio- or photosensitizer of DNA which could be used in cancer treatment therapy. In order to confirm the physicochemical properties of the system we conducted the series of experiments: chemical synthesis, DNase footprinting, DSC, CD, LC-MS analysis of oligonucleotide and molecular modeling.

The obtained stable complex of dsDNA with peptide mimicking the basic region of the GCN4 protein is a valuable model for DNA sensitization studies. In addition, the theoretical approach used for predicting the free energy of binding shows that theoretical insights into sensitization in systems for which DNA/protein interactions play a significant role is feasible. In the future, such approaches will promote a better understanding—and greater ease of predicting the properties—of new DNA sensitizers. Finally, this novel approach will fill the gap between currently used systems for studying DNA damage and will fit between pure oligonucleotides solutions and cell lines experiments allowing for tracing the nature of DNA damage upon radicals, solvated electrons, singlet oxygen, UV light and many others.

## Figures and Tables

**Table 1:** List of all abbreviation used and their respective sequences and calculated and experimental masses given by mass spectrometry.

Abbreviation	Composition	Experimental Mass [Da]	Theoretical Mass [Da]
PEP	H-ASP-PRO-ALA-ALA-LEU-LYS-ARG-ALA-ARG-ASN-THR-GLU-ALA-ALA-ARG-ARG-SER-ARG-ALA-ARG-LYS-GLY-GLY-LYS(N3)-NH2	2661.5001	2661.4320
ssDNA A	5′-GCA CGT CAT CCG TATAG-3′	5170.9401	5169.4020
ssDNA A*	5′-GCA XGT CAT CCG TATAG-3′ (X = C8-Alkyne dC)	5273.8241	5273.4591
ssDNA A**	5′-GCA XYT CAT CCG TATAG-3′ (X = C8-Alkyne dC, Y = 8-bromo-2′-deoxyguanosine)	5352.4231	5352.1046
ssDNA A*-PEP	5′-GCA XGT CAT CCG TATAG-3′ (X = PEP conjugated by CLICLK chemistry	7936.9349	7936.4653
ssDNA A**-PEP	5′-GCA XYT CAT CCG TATAG-3′ (X = PEP conjugated by CLICLK chemistry, Y = 8-bromo-2′-deoxyguanosine)	8013.9383	8013.4521
ssDNA B	5′-CTA TAC GGA TGA CGT GC-3′	5209.9135	5210.4530
dsDNA	ssDNA A: 5′-GCA CGT CAT CCG TATAG-3′ and ssDNA B: 5′-CTA TAC GGA TGA CGT GC-3′	-	-
dsDNA*	ssDNA A*: 5′-GCA XGT CAT CCG TATAG-3′ (X = C8-Alkyne dC) and ssDNA B: 5′-CTA TAC GGA TGA CGT GC-3′	-	-
dsDNA*-PEP	ssDNA A*-PEP: 5′-GCA XGT CAT CCG TATAG-3′ (X = PEP conjugated by CLICLK chemistry) and ssDNA B: 5′-CTA TAC GGA TGA CGT GC-3′	-	-
dsDNA*-PEP	ssDNA**-PEP: 5′-GCA XYT CAT CCG TATAG-3′ (X = PEP conjugated by CLICLK chemistry, Y = 8-bromo-2′-deoxyguanosine) and ssDNA B: 5′-CTA TAC GGA TGA CGT GC-3′	-	-

**Table 2:** Calculated thermodynamic contributions.

[kcal/mol]	ΔG_298_	ΔH_298_	ΔS_298_
dsDNA	17	101	0.28
dsDNA*-PEP	27	186	0.53

## Supplementary Materials

The following are available online, Figure S1: The detailed structure of the linker between oligonucleotide and peptide, R1—oligonucleotide residue, R2—peptide residue, Figure S2: Hydrogen bonding pattern of electrostatic interaction (green) along DNA (gray) and peptide (purple) sequences, Figure S3: High-resolution mass spectrum of ssDNA*-PEP after purification, Table S1: Table represent the identified by use of LC-MS fragments after DNase digestion and their representative peak areas. The peak areas where taken and plotted against the charge of the fragment of the oligonucleotide, Figure S4: Deconvolution of thermogram (orange line) of dsDNA in 100 mM PBS solution. Gray curves represent the deconvoluted states. The sum of the states is plotted in red, Figure S5: Deconvolution of thermogram (orange line) of dsDNA*-PEP in 100 mM PBS solution. Gray curves represent deconvoluted states. The sum of the states is plotted in red.
