# Supplementary material for: Generation and Characterization of a DNA-GCN4 Oligonucleotide-Peptide Conjugate: The Impact DNA/Protein Interactions on the Sensitization of DNA
Source: Molecules. 2020 Aug 10;25(16):3630. doi: 10.3390/molecules25163630 (PMC7466028; doi:10.3390/molecules25163630)
Supplement: Supplementary file 1 [file molecules-25-03630-s001.pdf]

molecules

Article

Generation and characterization of a DNA-GCN4 oligonucleotide-peptide conjugate: the impact DNA/protein interactions on the sensitization of DNA

Paweł Wityk [1,2,*], Rafał Piątek [2], Robert Nowak [3] and Dorota Kostrzewa-Nowak [3]

[1] Faculty of Chemistry, University of Gdańsk, Wita Stwosza 63, 80-308 Gdańsk, Poland
[2] Faculty of Chemistry, Gdańsk University of Technology, Narutowicza 11/12, 80-233 Gdańsk, Poland
[3] Centre for Human Structural and Functional Research, University of Szczecin, 17C Narutowicza St., 70-240 Szczecin, Poland

* Correspondence: pawel.wityk@pg.edu.pl

Supplementary materials

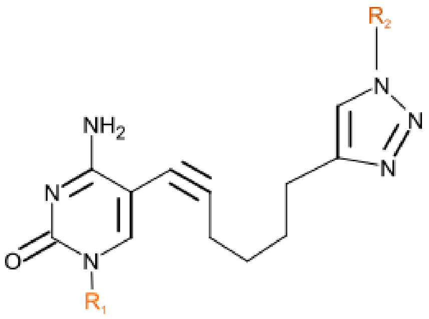

Figure S1. The detailed structure of the linker between oligonucleotide and peptide, R_1 – oligonucleotide residue, R_2 – peptide residue

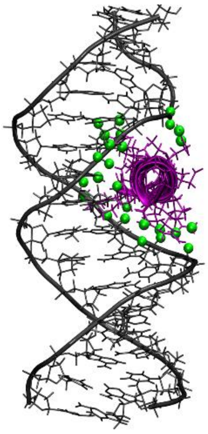

Figure S2. Hydrogen bonding pattern of electrostatic interaction (green) along DNA (gray) and peptide (purple) sequences.

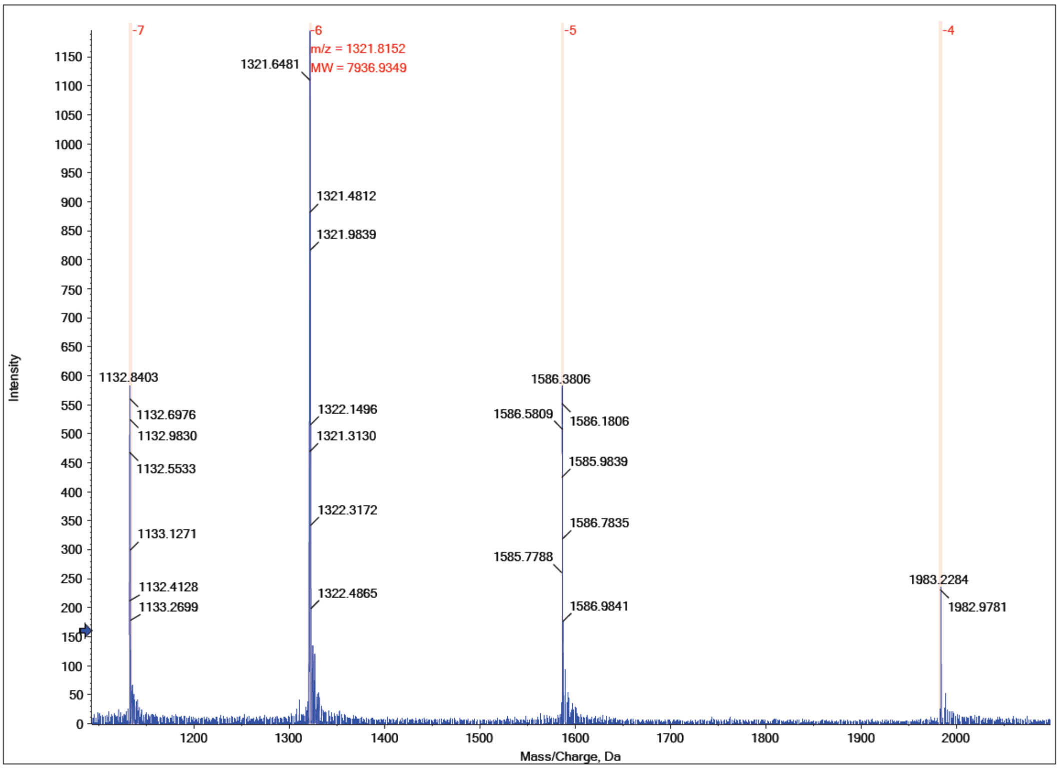

Figure 3. High-resolution mass spectrum of ssDNA*-PEP after purification.

Table S1. Table represent the identified by use of LC-MS fragments after DNase digestion and their representative peak areas. The peak areas where taken and plotted against the charge of the fragment of the oligonucleotide.

					Area [counts]	
	**Ret. Time [min]**	**Strand**	**Mass [Da]**	**Fragment**	**dsDNA**	**dsDNA-PEP**
**1**	22.667	A	5170.94	whole		
**2**	23.309	B	5210.94	whole	27100	29380
**3**	21.687	A	4527.6961	b15		
**4**	21.412	B	4591.7221	b15	164000	191700
**5**	21.2	A	3700.3406	w12		
**6**	21.091	A	4223.5841	b14		
**7**	20.605	B	4287.6425	b14	6271000	6184000
**8**	20.169	A	3396.2649	w11		
**9**	19.988	B	3766.3658	w12	27410	547800
**10**	19.285	B	3669.4163	b12	6238	12370
**11**	19.061	A	3606.3835	b12		
**12**	18.547	A	2754.0029	w11-b15		
**13**	18.115	A	2794.0151	w9		
**14**	17.933	B	3356.2966	b11	7919000	11980000
**15**	17.933	A	3277.2299	b11		
**16**	16.775	B	2844.0541	w12-b14	6812000	941500
**17**	16.619	A	2988.1572	b10		

**18**	16.276	A	2488.9088	w8		
**19**	15.58	B	2529.9208	w9-b16	347900	74510
**20**	15.25	B	2505.9057	x8	38100	342100
**21**	14.909	B	2723.0459	b9	3780000	1778000
**22**	14.317	A	2136.778	w14-b10/w13-b11		
**23**	13.247	A/B	2200.808	w7/w14-b10/w9-b15	10420000	5134000
**24**	11.11	A	2394.9209	b8		
**25**	11.11	B	2394.9209	w12-b12/w11-b13/w10-b14	25210000	50230
**26**	10.322	A	1910.6979	w6		
**27**	10.322	B	1871.6843	w6	620000	5566
**28**	9.511	B	2079.8061	b7	26490000	3170000
**29**	8.865	A	1580.5661	w5		
**30**	8.154	B	1582.5848	w6-b16/w7-b15/w8-b14/w12-b10/w14-b8/	16840000	3184000
**31**	7.95	B	1750.6962	b6	9316000	1451000
**32**	7.95	B	1558.5694	w5	1185000	1469000
**33**	7.613	A	1791.7047	b6		
**34**	6.861	A	1229.4513	w14-b7/w9-b12/w8-b13		
**35**	6.498	B	1462.5953	b5	18050000	3575000
**36**	5.225	A	1488.6024	b5		
**37**	4.943	A/B	924.3442	w-ACT-b	628700	16150
**38**	4.547	A/B	949.3552	w-ACG-b		
**39**	4.162	A/B	964.3576	w-AGT-b		
**40**	3.88	A/B	940.3425	w-CGT-b	1097000	128100
**41**	2.957	A/B	964.3624	w-AGT-b		
**42**	2.787	A/B	940.3425	w-CGT-b		
**43**	1.715	A/B	682.1904	w-AC-c		
**44**	1.7-0	A/B	All other fragments			

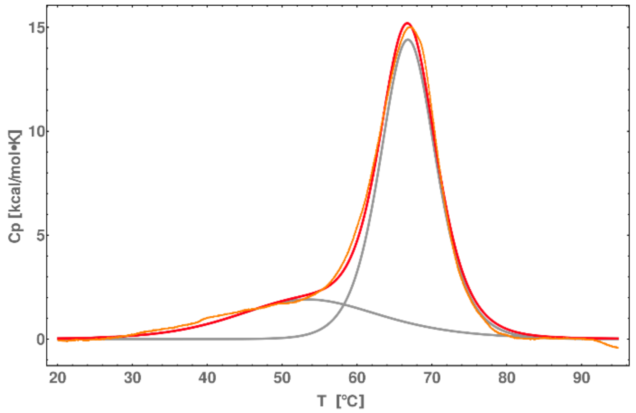

Figure S4. Deconvolution of thermogram (orange line) of dsDNA in 100 mM PBS solution. Gray curves represent the deconvoluted states. The sum of the states is plotted in red.

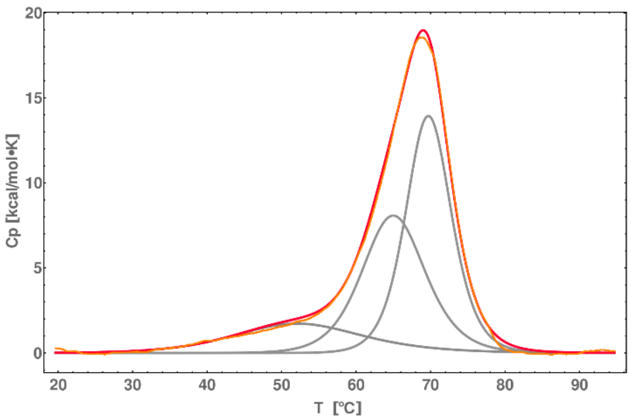

Figure S5. Deconvolution of thermogram (orange line) of dsDNA*-PEP in 100 mM PBS solution. Gray curves represent deconvoluted states. The sum of the states is plotted in red.
